# An AlGaN/GaN Lateral Bidirectional Current-Regulating Diode with Two Symmetrical Hybrid Ohmic-Schottky Structures

**DOI:** 10.3390/mi13071157

**Published:** 2022-07-21

**Authors:** Yijun Shi, Zongqi Cai, Yun Huang, Zhiyuan He, Yiqiang Chen, Liye Cheng, Guoguang Lu

**Affiliations:** National Key Laboratory of Science and Technology on Reliability Physics and Application of Electronic Component, China Electronic Product Reliability and Environmental Testing Research Institute, Guangzhou 510610, China; syj20094870@sina.com (Y.S.); huangyun@ceprei.com (Y.H.); yiqiang-chen@hotmail.com (Y.C.); q1238199@163.com (L.C.); luguoguang@ceprei.com (G.L.)

**Keywords:** current-regulating diode, hybrid-trench electrode, AlGaN/GaN, B-CRD

## Abstract

Bidirectional current-regulating ability is needed for AC light emitting diode (LED) drivers. In previous studies, various rectifier circuits have been used to provide constant bidirectional current. However, the usage of multiple electronic components can lead to additional costs and power consumption. In this work, a novel AlGaN/GaN lateral bidirectional current-regulating diode (B-CRD) featuring two symmetrical hybrid-trench electrodes is proposed and demonstrated by TCAD Sentaurus (California USA) from Synopsys corporation. Through shortly connecting the Ohmic contact and trench Schottky contact, the unidirectional invariant current can be obtained even with the applied voltage spanning a large range of 0–200 V. Furthermore, with the combination of two symmetrical hybrid-trench electrodes at each side of the device, the proposed B-CRD can deliver an excellent steady current in different directions. Through the TCAD simulation results, it was found that the device’s critical characteristics (namely knee voltage and current density) can be flexibly modulated by tailoring the depth and length of the trench Schottky contact. Meanwhile, it was also demonstrated through the device/circuit mixed-mode simulation that the proposed B-CRD can respond to the change in voltage in a few nanoseconds. Such a new functionality combined with excellent performance may make the proposed B-CRD attractive in some special fields where the bidirectional current-limiting function is needed.

## 1. Introduction

As a representative of wide-bandgap semiconductors, gallium nitride (GaN) has attracted increasing interest in high-voltage and high-power applications, due to its high critical electric field strength, high electron mobility, high saturation electron drift velocity, and high thermal conductivity [1]. The AlGaN/GaN high electron mobility transistor (HEMT) and metal-insulator-semiconductor HEMT (MIS-HEMT) are the most studied GaN-based power transistors, due to their high breakdown voltage, high switching frequency, low specific on-resistance, and especially the polarization-induced high-mobility and high-density two-dimensional electron gases (2DEG) within the AlGaN/GaN heterointerface [2]. Meanwhile, two-terminal power electronics are also indispensable components in a power electronic system, such as AlGaN/GaN lateral Schottky barrier diodes [3], AlGaN/GaN lateral field-effect rectifiers [4] and current-regulating diodes (CRD) [5,6]. Similar to the constant-current source based on integrated circuits, CRDs capable of providing a unidirectional invariant current are widely used in many power electronic systems, such as light emitting diode (LED) lighting systems and current-source systems for piezoelectric actuators.

Traditional circuits used to drive LEDs can be divided into switching constant-current modes and linear constant-current modes [7,8,9], which compose of multiple devices. In a typical linear constant-current driver circuit for an LED, N-Metal-Oxide-Semiconductor (NMOS), as the main power device, is connected in series with the LED and works in the linear amplification region. Additionally, the driving current of the LED can be adjusted by changing the gate voltage of NMOS. Another typical linear driver circuit for LEDs is the mirror constant-current circuit. The main power device, NMOS, also works in the linear amplification region. In this way, the current generated by the constant-current circuit will flow through the mirror circuit composed of two power devices to transfer a stable current to the LED, so that the driving current for the LED remains unchanged. In the application of medium- and small-power situations, a linear constant-current driver circuit has a low cost, a simple structure, a high efficiency, a small volume and low electromagnetic interference. It is also very suitable for indoor LED lamps (such as LED fluorescent lamps). However, a linear constant-current driver circuit is less used in high-power situations because of its low efficiency [10]. Different from the linear constant-current mode, power devices in switching constant-current circuits work in a high-speed switching state, which is controlled by switching power supply technology, so that the circuit can have a constant output current. However, a switching constant-current circuit is complex and requires a large board size. Based on the working characteristics of LEDs and the development trend of miniaturization in the semiconductor industry, LED driver circuits will be designed in a more simplified direction, and LED constant-current driver ICs will be more integrated. Constant-current driver circuits composed of CRDs are becoming more and more popular in the market. Compared with constant-current sources based on integrated circuits, CRDs are preferred due to their simple structure, high reliability, and strong anti-interference ability. In addition, they can protect LEDs from damage caused by a change in over-current, over-voltage and cycle number.

Although AlGaN/GaN CRDs are scarcely reported, developing a high-performance power electronic system based on AlGaN/GaN CRDs is of great potential. For example, high-performance single-chip LED lighting systems could be achieved through monolithically integrating AlGaN/GaN CRDs with GaN-based LEDs [11,12]. Furthermore, owing to their wide bandgap, AlGaN/GaN CRDs are also preferred for battery charge/discharge in harsh environments. We have proposed and experimentally investigated an AlGaN/GaN CRD based on a single hybrid Ohmic-Schottky structure [13]. Through shortly connecting the Ohmic contact and trench Schottky contact, the invariant current could be obtained for the proposed AlGaN/GaN CRD even with an applied voltage spanning a large range of 0–200 V and a wide temperature range from −50 °C to 250 °C. Meanwhile, the proposed AlGaN/GaN CRD exhibited a fast response capability. However, the proposed AlGaN/GaN CRD was only capable of providing an invariant current in one direction, and cannot provide an invariant current in another direction. CRDs with the capability of providing bidirectional invariant current is also needed in some application fields, such as AC LED lighting systems, battery charging/discharging systems, solar photovoltaic power generation systems, and energy management systems of electric vehicles [14,15]. In previous studies, various rectifier circuits have been to provide bidirectional constant current [16,17,18]. However, the usage of multiple electronic components can lead to additional costs and power consumption.

In this work, a novel AlGaN/GaN lateral bidirectional current-regulating diode (B-CRD) is proposed and demonstrated by TCAD Sentaurus. With the combination of two symmetrical hybrid-trench electrodes at each side of the device, the proposed B-CRD could deliver an excellent steady current in different directions. Furthermore, for the case of saving manpower, material resources, and time costs, the TCAD simulation was employed to investigate the proposed AlGaN/GaN B-CRD. The manuscript is organized as follows: the structure and mechanism of the proposed AlGaN/GaN B-CRD are briefly introduced in Part II; the device’s characteristics and their dependence on the thickness and length of AlGaN under the trench Schottky contact region (*T*_AlGaN_ and *L*_G_) are investigated in Part III; the conclusion is given in Part IV.

## 2. Structure and Mechanism

**Figure 1:**
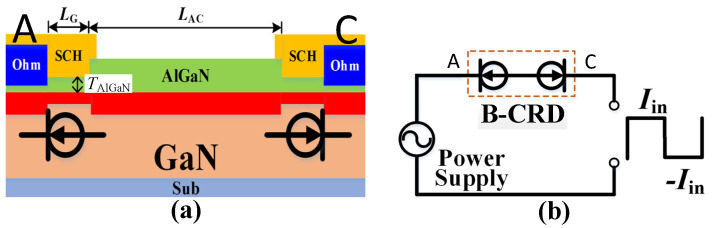
Schematic cross-section (**a**) and equivalent circuit (**b**) of the proposed AlGaN/GaN lateral B-CRD with two symmetrical hybrid-trench electrodes at each side of the device.

**Figure 2:**
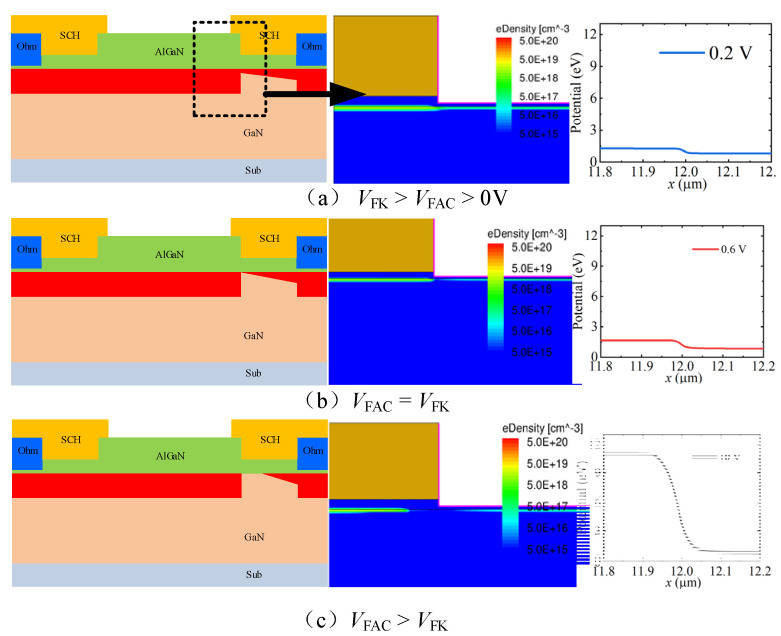
The forward working mechanism of the proposed AlGaN/GaN lateral B-CRD: (**a**) *V*_FK_ > *V*_FAC_ > 0 V; (**b**) *V*_FAC_ = *V*_FK_; (**c**) *V*_FAC_ > *V*_FK_.

**Figure 3:**
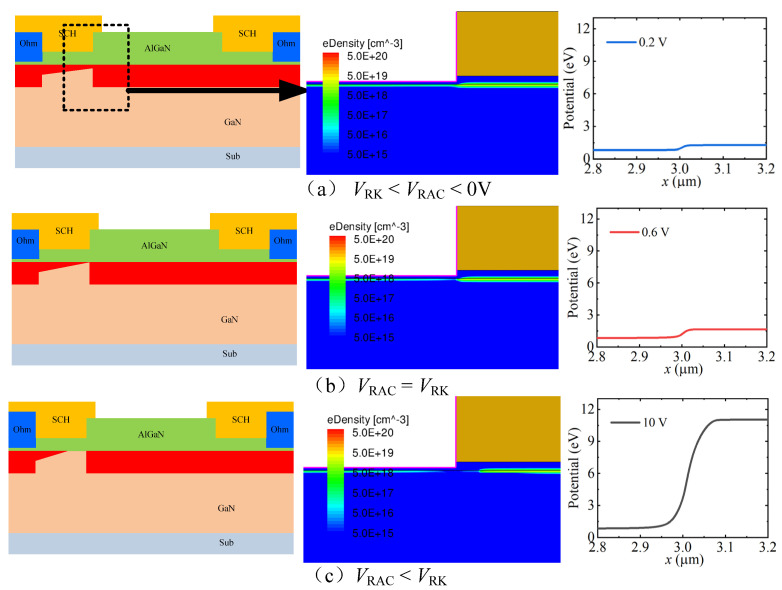
The reverse working mechanism of the proposed AlGaN/GaN lateral B-CRD: (**a**) *V*_FK_ > *V*_FAC_ > 0 V; (**b**) *V*_FAC_ = *V*_FK_; (**c**) *V*_FAC_ > *V*_FK_.

Figure 1 shows the schematic cross-section and equivalent circuit of the proposed AlGaN/GaN lateral B-CRD, which features two symmetrical hybrid-trench electrodes at each side of the device (Figure 1a). Both hybrid-trench electrodes consisted of a recessed Schottky contact structure and an Ohmic contact structure (Sch-A/C and Ohm-A/C, the left electrode is defined as the anode, the right electrode is defined as the cathode). The short connection of the recessed Schottky contact structure and Ohmic contact structure was able to provide an invariant current even with the applied voltage spanning a large range of 0–200 V. So, every hybrid-trench electrode could be treated as a unidirectional AlGaN/GaN lateral CRD, just as the experiment results in our previous work demonstrate [13]. The equivalent circuit of the unidirectional AlGaN/GaN lateral CRD is drawn under the recessed Schottky-contact structure (Figure 1a). For the proposed AlGaN/GaN lateral B-CRD, the hybrid-trench electrode at each side of the device could be regarded as a unidirectional AlGaN/GaN lateral CRD (Figure 1b). With the anti-series connection of two unidirectional CRDs, the proposed AlGaN/GaN lateral B-CRD was able to deliver an excellent steady current in different directions. The detailed working mechanism of the proposed AlGaN/GaN lateral B-CRD is explained in Figure 2 and Figure 3.

In the forward conduction state, the voltage drop was concentrated under the Sch-C region, which depleted the 2DEG under the Sch-C region, as shown in Figure 2. When the forward anode-to-cathode voltage (*V*_FAC_) was less than the forward-knee voltage (*V*_FK_), there was still a lot of two-dimensional electron gas (2DEG) under the Sch-C region, as shown in Figure 2a. In this stage, the forward anode-to-cathode current increased with the increase in the forward anode-to-cathode voltage, which also lead the 2DEG under the Sch-C region to be gradually depleted. When the forward anode-to-cathode voltage was equal to the forward knee voltage, the 2DEG channel was pinched off at the left side of the Sch-C region (as shown in Figure 2b), and a further increase in the forward anode-to-cathode voltage lead to the depletion area moving under the Sch-C region (as shown in Figure 2c). At this moment, the forward anode-to-cathode current did not continue to be increased with an increase in the forward anode-to-cathode voltage. The forward knee voltage and the forward constant-current density as the critical characteristics of the proposed AlGaN/GaN lateral B-CRD were determined by the depth and length of the trench Sch-C region. With the recessed Sch-C structure embedded into the AlGaN barrier layer, the depletion region under the recessed Schottky contact structure could be formed under a very low forward anode-to-cathode voltage, enabling the forward anode-to-cathode current density to become constant quickly. 

The same thing happened in the reverse conduction state. In the reverse conduction state, the voltage drop was concentrated under the Sch-A region, which lead to the depletion of the 2DEG under the Sch-A region, as shown in Figure 3. When the reverse anode-to-cathode voltage (*V*_RAC_) was less than the reverse knee voltage (*V*_RK_), the reverse anode-to-cathode current increased with an increase in the reverse anode-to-cathode voltage, due to there still being a lot of 2DEG under the Sch-A region, as shown in Figure 3a. When the reverse anode-to-cathode voltage was equal to the reverse knee voltage, the 2DEG channel was pinched off at the right side of the Sch-A region (as shown in Figure 3b). With a further increase in the reverse anode-to-cathode voltage, the depletion area moved under the Sch-A region (as shown in Figure 3c), and the reverse anode-to-cathode current did not continue to be increased. The reverse knee voltage and the reverse constant-current density were determined by the depth and length of the trench Sch-A region. With the recessed Sch-A structure embedded into the AlGaN barrier layer, the depletion region under the recessed Schottky contact structure could be formed under a very low reverse anode-to-cathode voltage.

In this work, TCAD Sentaurus was used to investigate the characteristics of the proposed AlGaN/GaN lateral B-CRD. The main physical models used in the simulation included a polarization model, an impact ionization model, and a high-field velocity saturation model [19,20,21]. The specific description of these models can be found in [21]. The correctness of the physical models employed in the simulation has been preliminarily verified in our previous work [19]. Furthermore, the experimental results in our previous work [13] were further used to correct the physical models employed in the simulation. The heterostructure employed in this work consisted of a 2 nm GaN cap, 23 nm Al_0.23_Ga_0.77_N barrier, 1 nm AlN spacer, and 3.5 μm GaN buffer, which was same as the heterostructure in our previous work [13]. In addition, the deep acceptor traps and self-compensating donor traps were also considered in the GaN buffer layer, with activation energies of *E*_V_ + 0.9 eV and *E*_C_ − 0.11 eV, and the trap densities were 3 × 10^16^ cm^−3^ and 1.3 × 10^15^ cm^−3^, respectively. Additionally, the unintentional doping concentration was set to be 1 × 10^15^ cm^−3^. The donor traps in the Nitride/GaN cap were considered with an activation energy of *E*_C_ − 0.4 eV and a density of 3 × 10^1^^3^ cm^−2^. In the beginning, the simulation results for the AlGaN/GaN lateral CRD with *T*_AlGaN_ = 9 nm and *L*_G_ = 1 μm were used to fit the experimental results to prove the correctness of the simulation. Then, the dependence of the proposed AlGaN/GaN lateral B-CRD’s characteristics on the depth and length of the trench Schottky contact were investigated. Finally, the device/circuit mixed-mode simulation was conducted to obtain the switching characteristics of the proposed AlGaN/GaN lateral B-CRD.

## 3. Results and Discussion

### 3.1. Static Characteristics of the Proposed AlGaN/GaN Lateral B-CRD

**Figure 4:**
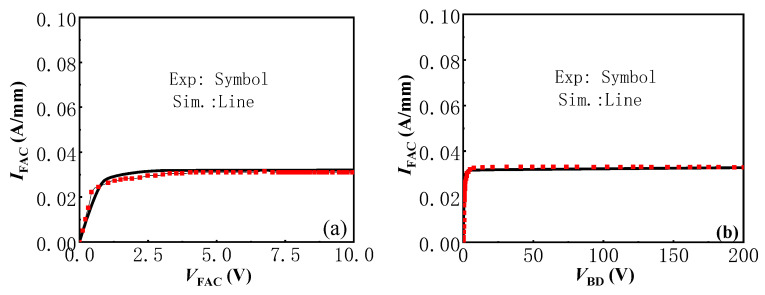
The *I*–*V* (**a**) and *BV* (**b**) characteristics of the AlGaN/GaN lateral CRD with *L*_G_ = 1 μm and *T*_AlGaN_ = 9 nm. Our pervious experimental results of the AlGaN/GaN lateral CRD in [13] are also presented for comparison.

Figure 4 shows the simulated current-voltage (*I*–*V*) and breakdown voltage (*BV*) characteristics of the AlGaN/GaN lateral CRD with *L*_G_ = 1 μm and *T*_AlGaN_ = 9 nm, as well as our pervious experimental results in [13]. It can be seen from the figure, there was no significant difference between the simulated characteristics of the AlGaN/GaN lateral CRD and the experimental results, which proves the correctness of the physical models employed in this research. In the following simulation work, the same physical models were employed to investigate the characteristics of the proposed AlGaN/GaN lateral B-CRD and their dependence on the depth and length of the trench Schottky contact.

**Figure 5:**
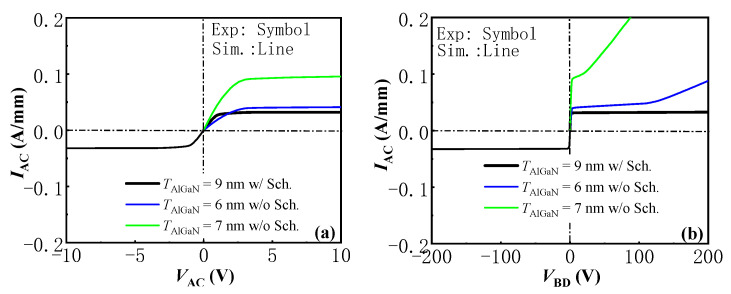
The simulated bidirectional *I*–*V* (**a**) and *BV* (**b**) characteristics of the proposed AlGaN/GaN lateral B-CRD with *L*_G_ = 1 μm and *T*_AlGaN_ = 9 nm, as well as with the simulated *I*–*V* and *BV* characteristics of the bidirectional diode without the Schottky electrode in the recessed structure.

**Figure 6:**
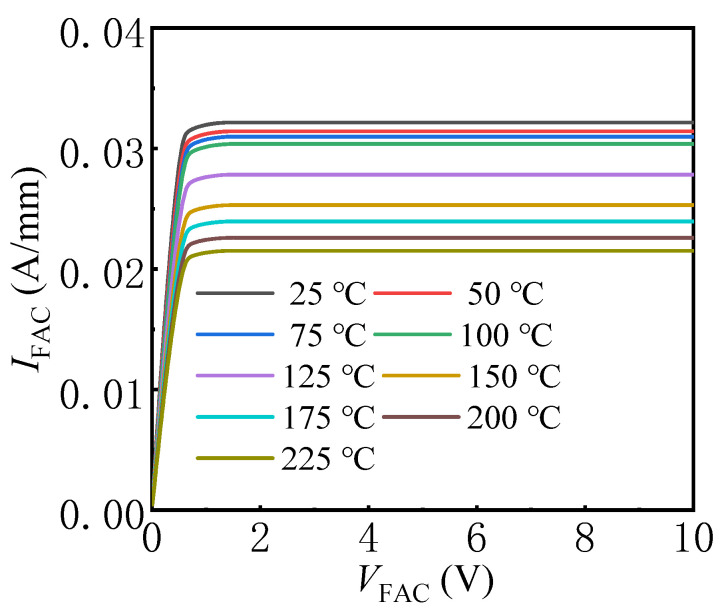
The influence of the temperature on the *I–V* characteristics of the proposed AlGaN/GaN lateral B-CRD.

Figure 5 exhibits the simulated bidirectional *I*–*V* and *BV* characteristics of the proposed AlGaN/GaN lateral B-CRD with *L*_G_ = 1 μm and *T*_AlGaN_ = 9 nm, as well as the simulated *I*–*V* and *BV* characteristics of the bidirectional diode without the Schottky electrode in the recessed structure. As stated above, the proposed AlGaN/GaN lateral B-CRD was able to deliver an excellent steady current in not only the forward conduction state, but also the reverse conduction state. Furthermore, compared with the CRD in [5,6], the proposed AlGaN/GaN lateral B-CRD delivered a steadier current even with the applied voltage spanning a large range of 0–200 V. Additionally, the knee voltage of the proposed AlGaN/GaN lateral B-CRD was about 0.6 V, which was much lower than that of the CRD in [5,6] (about 3 V). Such a new functionality combined with the excellent performance may make the proposed AlGaN/GaN lateral B-CRD attractive in some special fields, where a bidirectional current-limiting function is needed. In Figure 4, it was also found that the bidirectional diode without the Schottky electrode in the recessed structure could not deliver an invariant current with the applied voltage spanning a range of 0–200 V, which demonstrates that the Schottky contact electrode in the recessed structure played an important role in delivering an excellent steady current for the proposed AlGaN/GaN lateral B-CRD. The influence of the temperature on the *I–V* characteristics of the proposed AlGaN/GaN lateral B-CRD was also investigated, as shown in Figure 6. It can be seen that the current of the proposed AlGaN/GaN lateral B-CRD decreased with an increase in temperature, which was due to the decrease in electron mobility. As the temperature increased from 25 °C to 225 °C, the current of the proposed AlGaN/GaN lateral B-CRD decreased from 32 mA to 22 mA, a decrease of about 30%. However, it can be seen that the increase in the temperature had nearly no effect on the knee voltages of the proposed AlGaN/GaN lateral B-CRD. The highly stable knee voltages may make the proposed AlGaN/GaN lateral B-CRD more attractive.

**Figure 7:**
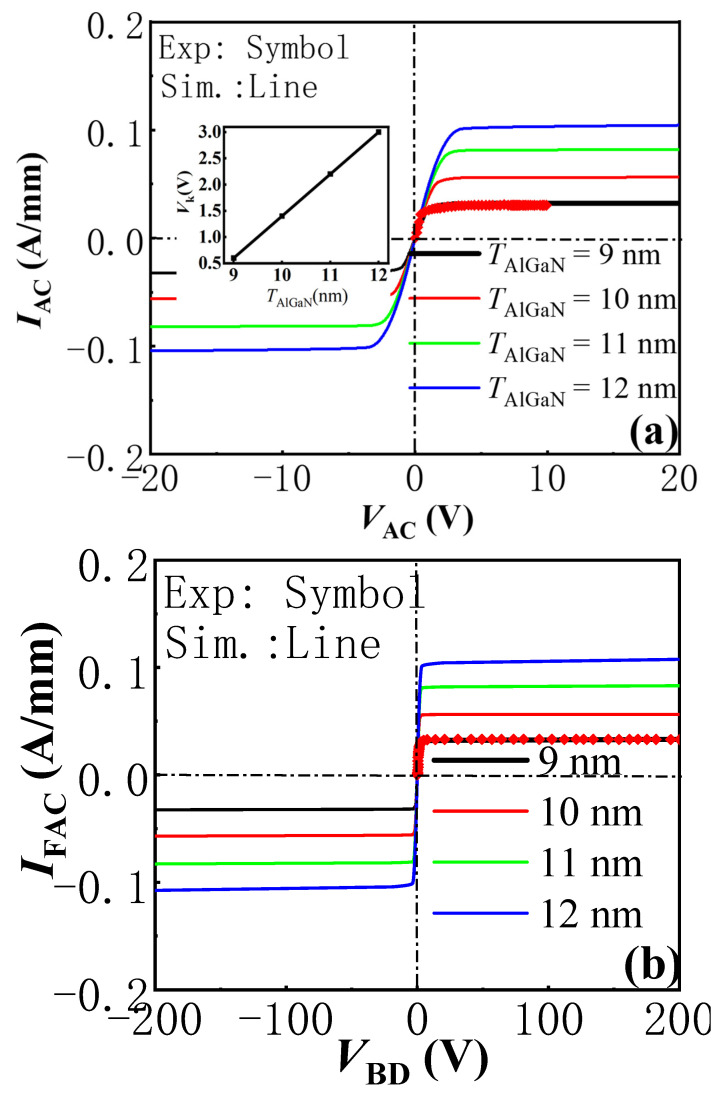
The simulated bidirectional *I*–*V* (**a**) and *BV* (**b**) characteristics of the proposed AlGaN/GaN lateral B-CRD with *L*_G_ = 1 μm and different *T*_AlGaN_.

As mentioned, the knee voltage and constant-current density of the proposed AlGaN/GaN lateral B-CRD were determined by the depth and length of the trench Schottky contact. So, for helping the actual device design, it is necessary to investigate the device characteristic’s dependence on the depth and length of the trench Schottky contact. Figure 7 gives the influence of the trench Schottky contact’s depth on the bidirectional *I*–*V* and *BV* characteristics of the proposed AlGaN/GaN lateral B-CRD with *L*_G_ = 1 μm. It was found that the constant current density of the proposed AlGaN/GaN lateral B-CRD increased with an increase in *T*_AlGaN_. When *T*_AlGaN_ was increased from 9 nm to 12 nm, the constant-current densities increased from 32 mA to 100 mA (an increase of more than 200%). Although the proposed AlGaN/GaN lateral B-CRD with a large *T*_AlGaN_ was able to deliver a higher current density, the pronounced self-heating effect may significantly deteriorate the steadiness of the regulating current as well as the effective operating voltage range, as shown in our pervious experimental results [13]. On the other hand, by reducing *T*_AlGaN_, the gate-to-channel controllability was effectively enhanced due to the smaller gate-to-channel distance, which lead to a much lower knee voltage (the inset in Figure 7) and an improved steadiness in the regulating current. As can also be seen from the figure, as *T*_AlGaN_ decreased from 12 nm to 9 nm, the knee voltages decreased from 3 V to 0.6 V. Additionally, the power consumption (*V*_K_*I*_AC_) of the proposed AlGaN/GaN lateral B-CRD could be respectably reduced. So, through tailoring the depth of the trench Schottky contact, a desirable knee voltage and constant-current density could be obtained for the proposed AlGaN/GaN lateral B-CRD.

**Figure 8:**
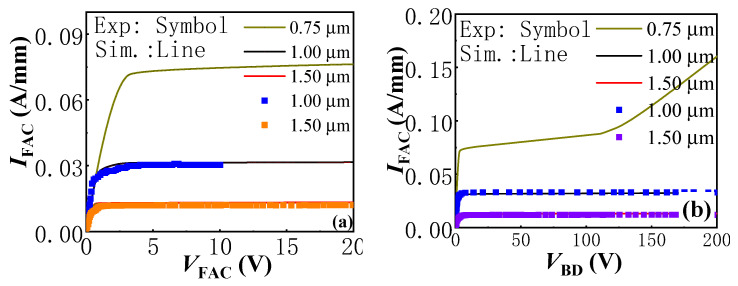
The simulated bidirectional *I*–*V* (**a**) and *BV* (**b**) characteristics of the proposed AlGaN/GaN lateral B-CRD with *T*_AlGaN_ = 9 nm and different *L*_G_.

In this part, the influence of the trench Schottky contact length on the bidirectional *I*–*V* and *BV* characteristics of the proposed AlGaN/GaN lateral B-CRD is investigated, as shown in Figure 8. The value of *T*_AlGaN_ of the trench Schottky contact was set to be 9 nm for the proposed AlGaN/GaN lateral B-CRD. It can be seen that the constant-current densities of the proposed AlGaN/GaN lateral B-CRD increased with a decrease in *L*_G_. As *L*_G_ decreased from 1.5 μm to 0.75 μm, the constant-current densities increased from 13 mA to 75 mA. Although the proposed device with a short *L*_G_ was able to deliver a higher current density, the device also possessed low breakdown voltage (Figure 8b), which may be due to the short-channel effect. The decrease in the trench Schottky contact length lead to a significant reduction in barrier thickness, which made the electrons more easily transmissible, subsequently reducing the breakdown voltage of the proposed AlGaN/GaN lateral B-CRD. So, to enable the proposed AlGaN/GaN lateral B-CRD with a stable regulating current and a high breakdown voltage, the trench Schottky contact length should be long enough.

### 3.2. Switching Characteristics of the Proposed AlGaN/GaN Lateral B-CRD

**Figure 9:**
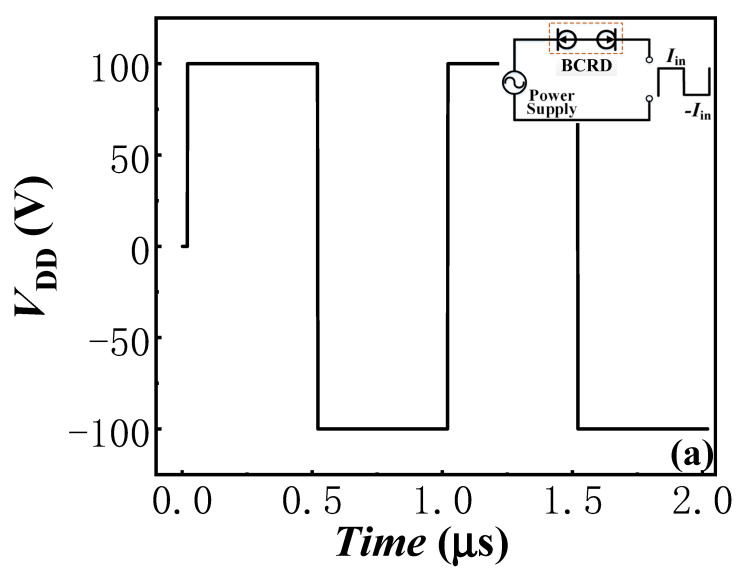

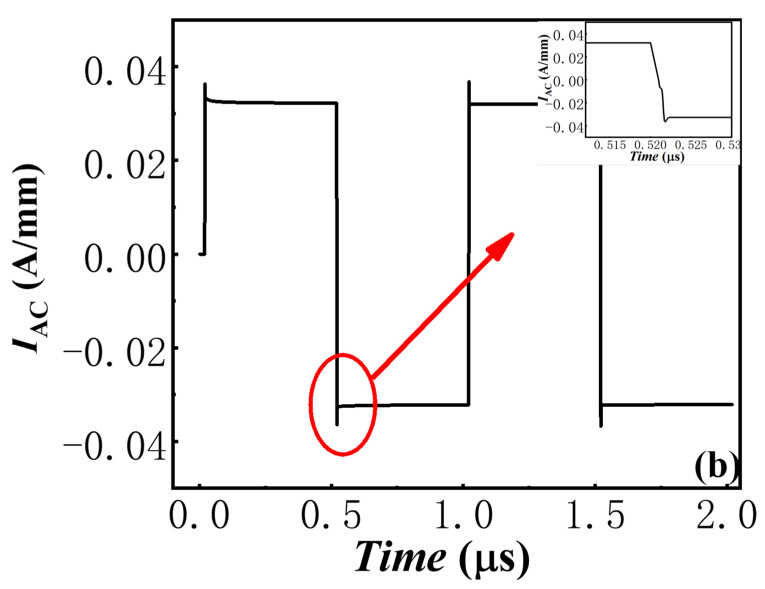
The switching characteristics of the proposed AlGaN/GaN lateral B-CRD: (**a**) applied voltage; (**b**) current.

Besides outstanding static characteristics, excellent switching characteristics are also important for two-terminal power rectifiers. In this work, the circuit in Figure 1b was employed to evaluate the switching characteristics of the proposed AlGaN/GaN lateral B-CRD. The depth and length of the trench Schottky contact of the proposed AlGaN/GaN lateral B-CRD were set to be 9 nm and 1 μm, respectively. As shown above, the proposed AlGaN/GaN lateral B-CRD with *T*_AlGaN_ of 9 nm and *L*_G_ of 1 μm possessed a low constant-current density of about 30 mA/mm, even with the applied voltage spanning a range of 0–200 V. In addition, two applied voltages with different directions were employed in this circuit (Figure 9a), which were set to be 100 V and −100 V. In addition, the switching time between the two applied voltages was set to 1 ns.

**Figure 10:**
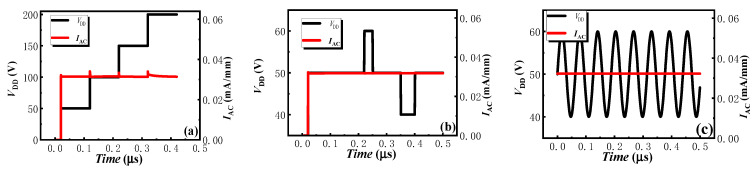
The anti-interference ability of the proposed AlGaN/GaN lateral B-CRD: (**a**) 50 V to 200 V, (**b**) change from 50 V to 60 V or to 40 V, (**c**) change in sine function.

Figure 9b exhibits the switching characteristics of the proposed AlGaN/GaN lateral B-CRD. When a high positive voltage of 100 V was applied to the proposed AlGaN/GaN lateral B-CRD, the device maintained a relatively low and stable forward current of about 30 mA/mm. When the switch was turned to a negative applied voltage, the proposed AlGaN/GaN lateral B-CRD produced a reverse-current overshoot and a high current change rate, as shown in Figure 9b. The current overshoot may result from the reverse-recovery charge of the hybrid-trench electrodes, just as the reverse-recovery phenomenon in the AlGaN/GaN lateral-field-effect rectifiers [19]. However, there were very little reverse-recovery charges for the hybrid-trench electrodes. So, the reverse-current overshoot of the proposed AlGaN/GaN lateral B-CRD was very low, and the proposed B-CRD was able to respond quickly to the change in the applied voltage and reached a steady state in a few nanoseconds, even with a very high change rate in the applied voltage. In other words, the proposed AlGaN/GaN lateral B-CRD possessed an excellent switching characteristic. Additionally, the anti-interference ability of the proposed AlGaN/GaN lateral B-CRD was also evaluated by the circuit shown in Figure 1. The applied voltages were set to increase from 50 V to 200 V (Figure 10a), change from 50 V to 60 V or to 40 V (Figure 10b), or change in sine function (Figure 10c). The switching time between the different voltages was also set to 1 ns. Figure 8 exhibits the anti-interference ability of the proposed AlGaN/GaN lateral B-CRD. It was found that the sudden change in the applied voltage had nearly no effect on the current of the proposed AlGaN/GaN lateral B-CRD, demonstrating the proposed B-CRD possessed an excellent anti-interference ability.

## 4. Conclusions

A novel AlGaN/GaN lateral B-CRD featuring two symmetrical hybrid-trench electrodes is proposed in this paper. Through the simulation carried out by TCAD Sentaurus, it was demonstrated that the proposed B-CRD was able to deliver an excellent steady current in both directions, even with the applied voltage spanning a large range of 0–200 V. Furthermore, the device’s knee voltage and current density showed a strong relation with the depth and length of the trench Schottky contact. Through tailoring the depth and length of the trench Schottky contact, a desirable knee voltage and constant-current density could be obtained for the proposed AlGaN/GaN lateral B-CRD. Additionally, it was also demonstrated through the device/circuit mixed-mode simulation that the proposed B-CRD was able to respond to the change in voltage in a few nanoseconds.

## Data Availability

Data available on request due to restrictions e.g., privacy or ethical.
